# Supplementary figures and images for: A Mechanistic Model for Reward Prediction and Extinction Learning in the Fruit Fly
Source: eNeuro. 2021 Jun 15;8(3):ENEURO.0549-20.2021. doi: 10.1523/ENEURO.0549-20.2021 (PMC8211469; doi:10.1523/ENEURO.0549-20.2021)

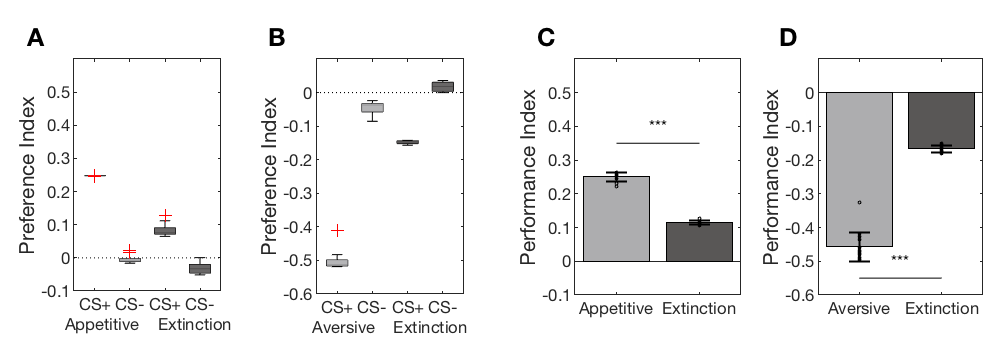

Supplement: Extended Data Figure 3-2 — Independent tuning of the model parameters for the appetitive and aversive pathways allows to match the experimental results more accurately. In order to fit the model to the asymmetry in the experimental results, the PPL1 output was tuned towards a lower activation threshold with the sigmoid transfer function PPL1=1(1+10000×e−PPL1Input×21). In consequence, lateral inhibition between MBONs was appropriately adjusted: MVP2::M6 inhibition was increased to M6–=−0.6(1+200×e(−MVP2×16)), MV2::V2 inhibition was decreased to V2−=−0.6(1+200×e(−MV2×13)). Further, the factor ρ representing the inhibition of the PAM DAN was set to 0.5 for the aversive learning conditioning paradigm. With the tuned model we can achieve a stronger aversive memory (B, D) that fits quantitatively the experimental results (Table 1). The tuned model also achieves a stronger extinction effect in the appetitive learning paradigm (A, C); however, it does not abolish appetitive memory completely. Boxplots show the median and the lower and upper quartiles, whiskers indicate 1.5 times interquartile range, outliers are marked with + symbol. Bar plots are presented as mean ± SD; n = 10 independent models Download Figure 3-2, TIF file. [file enu-eN-NWR-0549-20-s04.tif]
